# Short‐Term Isocaloric Intake of a Fructose‐ but not Glucose‐Rich Diet Affects Bacterial Endotoxin Concentrations and Markers of Metabolic Health in Normal Weight Healthy Subjects

**DOI:** 10.1002/mnfr.201800868

**Published:** 2019-01-02

**Authors:** Anika Nier, Annette Brandt, Dragana Rajcic, Tony Bruns, Ina Bergheim

**Affiliations:** ^1^ Department of Nutritional Sciences Molecular Nutritional Science University of Vienna 1090 Vienna Austria; ^2^ SD Model Systems of Molecular Nutrition Institute of Nutrition Friedrich–Schiller University Jena 07743 Jena Germany; ^3^ Department of Internal Medicine IV University Hospital Jena 07743 Jena Germany

**Keywords:** endothelial function, endotoxin, fructose, glucose, normal weight healthy adults

## Abstract

**Scope:**

Dietary pattern and impairments of intestinal barrier function are discussed to be critical in the development of metabolic impairments. Here, it is determined if an isocaloric exchange of complex carbohydrates with monosaccharides affects markers of intestinal permeability and metabolic health in healthy subjects.

**Methods and Results:**

After a dietary standardization for 4 days, all 12 subjects aged 21–33 years receive an isocaloric fructose‐ and glucose‐enriched diet for 3 days separated by a wash‐out phase. Anthropometry, blood pressure, markers of intestinal permeability and metabolic as well as inflammatory parameters are determined in blood samples or isolated peripheral blood mononuclear cells collected at baseline, after standardizations and the monosaccharide interventions, respectively. While anthropometric and inflammatory parameters are not changed, the intake of an isocaloric fructose‐ but not glucose‐enriched diet is associated with a significant increase of bacterial endotoxin plasma levels and alanine aminotransferase activity in serum, while total plasma nitrate/nitrite concentrations are significantly decreased. In peripheral blood mononuclear cells, *Toll like receptors 4, 2*, and *MYD88* mRNA expressions are significantly induced after the fructose‐rich but not the glucose‐rich diet.

**Conclusion:**

In metabolically healthy subjects, even a short‐term intake of a fructose‐rich diet can elevate bacterial endotoxin levels and change markers of liver health and vascular endothelial function.

## 1 Introduction

General overnutrition and physical inactivity have repeatedly been proposed to be among the key risk factors for many overweight‐associated metabolic diseases including nonalcoholic fatty liver disease (NAFLD) and cardiovascular diseases.^1^ In recent years, it has been discussed that dietary pattern may affect the development of NAFLD^2^ and diseases of the cardiovascular system.^3^ Indeed, results of several epidemiological studies suggest that a diet rich in red meat, fats, and sweets may promote the development of NAFLD and hypertension as well as vascular endothelial dysfunction in humans,^2^, ^4^ while the intake of whole grains, fruits, and vegetables were frequently found to be lower in patients with metabolic diseases than in disease free controls.^5^ In line with these findings, results of animal studies employing pair‐feeding models also suggest that consumption of fructose, particularly in combination with saturated fat, may be critical in the development of NAFLD.^6^ In rodents chronic intake of a fructose‐rich diet is associated with the development of vascular dysfunction and hypertension.^7^ In addition to chronic extended intake of a fructose‐ and/ or fat‐rich diet,^8^ even a short‐term isocaloric change to diets enriched in fructose or fructose‐ and fat can lead to the development of early signs of NAFLD and vascular endothelial dysfunction.^9^ These alterations are associated with a loss of tight junction proteins in small intestine and increased endotoxin concentrations in portal plasma.9a However, results of short‐term intervention studies in healthy humans show contradictory results regarding the effects of fructose on liver (for overview see ref. [10]), which might have resulted from marked differences in study design and duration while data on the effects on vascular endothelium in humans to our knowledge are lacking. Thus, the aim of the present study was to determine if an isocaloric 3‐day‐long exchange of complex carbohydrates with fructose or glucose affects surrogate markers of liver health and vascular endothelial function in healthy normal weight young male and female adults and if so, whether these effects are related to changes in markers of intestinal permeability like bacterial endotoxin and lipopolysaccharide binding protein (LBP).

## 2 Experimental Section

### 2.1 Study Participants

The present study was approved by the Institutional Review Board of the University Hospital Jena, Jena, Germany (4588‐11/15) and was carried out in accordance with the ethical standards laid down in the Declaration of Helsinki of 1975 as revised in 1983. The study is registered at ClinicalTrials.gov (NCT03482284). Based on sample size calculations, a total of 15 normal weight healthy subjects were enrolled in the study after giving written informed consent to participate in the study. As three participants dropped out due to intestinal discomfort, only 12 subjects were included in the final analysis. None of the participants had a known history of I) diabetes mellitus, II) elevated triglyceride concentrations, III) obesity, IV) elevated blood pressure, V) low HDL concentrations, VI) elevated waist circumference, or VII) liver diseases when enrolled in the study. None of the participants stated to smoke or to drink alcohol exceeding a moderate intake (>10 g d^–1^ for women; >20 g d^–1^ for men as defined by the National Institute on Alcohol Abuse and Alcoholism).^11^

### 2.2 Study Design

**Figure 1:**
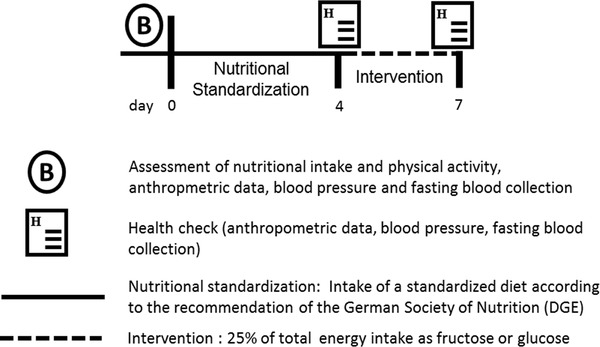
Study design.

As detailed in **Figure** 1, all participants received a standardized diet for 4 days, followed by a diet in which complex carbohydrates were either exchanged with fructose or glucose (25% of total energy intake) for 3 days. All participants analyzed consumed both sugar‐enriched diets after being dietary standardized for 4 days starting with the fructose intervention. Sugar interventions were separated by a washout period of at least 3 weeks. During the dietary standardization, participants were requested to follow a diet according to the recommendations of the German Society of Nutrition,^12^ which was adjusted to the individual caloric intake as assessed in two independent 24‐h recalls before the study (see below). During weekdays, foods and beverages consumed during the standardization period and the high fructose as well as high glucose intervention phases were provided, prepared, and consumed at the study center. To enhance compliance, participants were offered to prepare foods and beverages consumed on weekends at home, following recipes provided by the study team. Fructose and glucose for the fructose‐ and glucose‐enriched diet, respectively, were provided to all participants as jelly. Jellies were pre‐prepared by the study team and handed to the study participants freshly on each study day in three separated portions adjusted to caloric intake of the respective subject. Nutritional intake during standardization and the interventions is summarized in **Table** 1. During the wash out period all participants consumed their usual diet.

**Table 1:** Composition of the standard and intervention diets

Nutrient	Standard diet 1	Fructose rich diet	Standard diet 2	Glucose rich diet
Total energy (kcal d^–1^)	2629 ± 134.3	2619 ± 130.2	2616 ± 135.5	2619 ± 130.2
Total fat (%E)	30.4 ± 0.2	30.4 ± 0.2	31.3 ± 0.2	30.4 ± 0.2
Total protein (%E)	16.4 ± 0.4	15.9 ± 0.2	15.9 ± 0.3	15.9 ± 0.2
Total carbohydrate (%E)	53.1 ± 0.4	53.7 ± 0.2	52.7 ± 0.3	53.7 ± 0.2
Total fructose (g d^–1^)^a^	57.9 ± 2.9	178.0 ± 8.7* ^,^ ***	54.9 ± 3.2	18.9 ± 0.2* ^,^ ** ^,^ ***
Total glucose (g d^–1^)^b^	54.3 ± 2.6	16.3 ± 0.2* ^,^ ***	54.9 ± 3.2	175.4 ± 8.6* ^,^ ** ^,^ ***
Sucrose (g d^–1^)	65.3 ± 5.3	11.5 ± 0.2* ^,^ ***	59.9 ± 4.4	11.5 ± 0.2* ^,^ ***
Starch (g d^–1^)	200.9 ± 10.4	121.0 ± 7.8*, ,***	202.7 ± 10.8	121.0 ± 7.8* ^,^ ***
Fiber (g d^–1^)	36.3 ± 1.4	38.4 ± 1.0	37.4 ± 1.7	38.4 ± 1.0

Data are shown as mean ± SEM

a Free fructose and fructose deriving from sucrose

b Free glucose and glucose deriving from sucrose

*p < 0.05 in comparison to standard diet 1

**p < 0.05 in comparison to fructose rich diet

***p < 0.05 in comparison to standard diet 2.

### 2.3 Dosage Information

Individual daily total energy requirements of each participant were calculated using EBISpro. Based on these calculations, participants received a standardized diet according to their individual caloric needs and the recommendations of the German Society of Nutrition for 4 days, followed by a 3‐day high fructose and high glucose diet, respectively. During the interventions, fructose and glucose were provided as jellies prepared by the study team. Fructose and glucose, respectively accounted for 25% of the daily total energy intake with an average intake of ≈160 g d^–1^ and person.

### 2.4 Blood Sampling, Nutritional Intake, Physical Activity, Anthropometry, and Blood Pressure

Nutritional intake was assessed using two independent 24‐h recalls conducted by an experienced nutritionist before the study. To assess physical activity level, the Global Physical Activity Questionnaire established by the World Health Organization was used to determine physical activity of subjects (http://www.who.int/ncds/surveillance/steps/GPAQ/en/). Nutritional data and total energy expenditure were analyzed using the computer software EBISpro (Version 2011, Germany). Fasting blood samples, anthropometric data and blood pressure were assessed at the beginning of the study, after consuming the standard diets and after the intervention periods as described previously.^13^

### 2.5 Peripheral Blood Mononuclear Cell Isolation, RNA Extraction, and Real‐Time PCR

Peripheral blood mononuclear cells (PBMCs) were isolated from whole blood samples by density gradient separation. RNA was isolated from isolated PBMCs using Trizol (peqGOLD Trifast, Peqlab, Germany) and reverse transcribed using a cDNA synthesis kit (Promega, Germany) following the instructions of the manufacturer. Primers used to determine *toll like receptor (TLR)4*, *TLR2*, and *myeloid differentiation primary response gene* (*MYD)88* mRNA expression were designed using the software Primer 3. To determine the amount of target genes, normalized on endogenous reference (*18S*) and relative to a calibrator (2^−ΔΔCt^), the comparative C_T_ method was used as detailed previously.8d Primer sequences are shown in **Table** 2.

**Table 2:** Primer sequences

Gene	Forward (5´–3´)	Reverse (5´–3´)
*18S*	GGGCCCGAAGCGTTTACTTT	CGCCGGTCCAAGAATTTCAC
*TLR‐2*	ATTGTGCCCATTGCTCTTTC	CTGCCCTTGCAGATACCATT
*TLR‐4*	TGAGCAGTCGTGCTGGTATC	CAGGGCTTTTCTGAGTCGTC
*MyD88*	GCACATGGGCACATACAGAC	GACATGGTTAGGCTCCCTCA

TLR, toll‐like receptor; MyD88, myeloid differentiation primary response gene 88.

### 2.6 Endotoxin Analysis

To determine endotoxin concentration in plasma, samples were heated for 20 min at 70 °C as detailed before.^14^ Tween 80 (20%, Carl Roth, Germany) was added and samples were treated in an ultrasonic bath for 5 min. Endotoxin was measured using a commercially available kit (Charles River, Germany) based on *Limulus amebocyte lysate* (LAL). Recovery rates were on average around 125%.

### 2.7 ELISAs and NOx Measurement

Using commercially available ELISA kits, the following parameters were analyzed in plasma and serum, respectively: leptin (Hölzel GmbH, Germany), plasminogen activator inhibitor (PAI)‐1 (LOXO GmbH, Germany), LBP (Abnova, Taiwan), d‐lactate (Cayman Chemical, USA), l‐citrulline (CUSABIO, USA), adiponectin (TECOmedical AG, Switzerland), total nitrate/nitrite (NOx) (Cell Biolabs, Inc., USA), and endothelin (ET)‐1 (Cloud‐Clone Corp., USA).

### 2.8 Liver Transaminases and Blood Lipid Concentrations

Liver transaminases and blood lipid concentrations were analyzed by routine laboratory methods (University Hospital Friedrich‐Schiller‐University Jena).

### 2.9 Statistical Analysis

Results are presented as absolute numbers or as mean ± SEM. Data were analyzed using Wilcoxon test to compare values of the intervention with the values of the respective standardization. Differences in nutritional intake between the four different diets were analyzed using Friedman Test. All data were analyzed with GraphPad Prism (version 7.03, 2017, GraphPad Software Inc., USA). A *p*‐value < 0.05 was considered significant.

## 3 Results

### 3.1 Baseline Characteristics and Nutritional Standardization

Baseline characteristics including anthropometry, nutritional intake, and health related parameters of all participating subjects are summarized in **Table** 3. In total, 12 healthy normal weight subjects (seven women and five men) aged 21–33 years were analyzed as three subjects dropped out before finishing the study. After assessing energy and nutritional intake, all subjects consumed an isocaloric nutritionally balanced standard diet following the recommendations of 2015 of the German Nutritional Society.^12^ Body mass index (BMI), waist circumference, and blood pressure as well as most blood parameters remained unchanged during the 4 days of nutritional standardization when compared to baseline (Table 3). However, as for most subjects, the standard diet was associated with switching from a fat‐ to a fiber‐rich and carbohydrate‐rich diet, serum triglyceride levels (≈+1.3‐fold) as well as plasma insulin levels (≈+1.2‐fold) were increased significantly after standardization when compared to baseline while alanine aminotransferase (ALT) serum activity was significantly lower when compared to baseline (≈–7%). Furthermore, bacterial endotoxin levels (≈–19%) were also significantly lower after the dietary standardization while LBP, NOx, and ET‐1 levels remained unchanged (Table 3).

**Table 3:** Baseline characteristics of all study participants and after nutritional standardization

Parameter	Baseline	Nutritional standardization
*n*	12	
Sex (female/male)	7/5	
Age (years)	26.3 ± 1.2	
Weight (kg)	66.5 ± 3.3	66.5 ± 3.3
Waist circumference (cm)	71.2 ± 1.7	71.3 ± 1.7
BMI (kg m^–^²)	22.0 ± 0.7	22.0 ± 0.7
Total caloric intake (kcal d^–1^)	2459 ± 162.1	2629 ± 134.3
Total fat intake (%E)	40.2 ± 2.1	30.4 ± 0.2*
SFA (g d^–1^)	49.3 ± 4.4	38.9 ± 2.8*
MUFA (g d^–1^)	37.9 ± 3.2	31.2 ± 1.4
PUFA (g d^–1^)	15.8 ± 2.6	12.6 ± 0.6
Total protein intake (%E)	15.1 ± 0.8	16.4 ± 0.4
Total CHO intake (%E)	44.0 ± 1.9	53.1 ± 0.4*
Fructose (%E)a)	8.0 ± 0.9	9.0 ± 0.3
Glucose (%E)b)	7.3 ± 0.9	8.5 ± 0.2
Sucrose (%E)	9.0 ± 1.1	10.1 ± 0.4
Starch (%E)	24.8 ± 2.3	31.3 ± 0.3*
Fiber intake (g d^–1^)	22.6 ± 2.9	36.3 ± 1.4*
ALT (U L^–1^)	29.8 ± 3.2	27.8 ± 3.1*
AST (U L^–1^)	24.3 ± 1.8	23.4 ± 1.4
NOx (µm)	51.0 ± 6.3	49.0 ± 8.9
ET‐1 (pg mL^–1^)	21.2 ± 1.8	21.0 ± 1.4
Systolic blood pressure (mmHg)	123.4 ± 1.8	119.9 ± 1.6
Diastolic blood pressure (mmHg)	71.6 ± 2.7	68.7 ± 2.9
TAG (mg dL^–1^)	82.1 ± 10.2	105.8 ± 14.3*
Fasting glucose (mg dL^–1^)	89.8 ± 2.1	90.1 ± 1.6
Insulin (µIU mL^–1^)	10.3 ± 1.3	12.0 ± 1.1*
Uric acid (mg dL^–1^)	5.2 ± 0.3	5.0 ± 0.3
Endotoxin (EU mL^–1^)	0.32 ± 0.02	0.26 ± 0.02*
LBP (µg mL^–1^)	22.6 ± 2.8	21.3 ± 2.2

^a)^Free fructose and fructose deriving from sucrose; ^b)^Free glucose and glucose deriving from sucrose. Data are shown as absolute numbers and mean ± SEM, respectively. SFA, saturated fatty acids; MUFA, monounsaturated fatty acids; PUFA, polyunsaturated fatty acids; CHO, carbohydrate; ALT, alanine aminotransferase; AST, aspartate aminotransferase; NOx, Total nitrate/nitrite; ET‐1, endothelin‐1; TAG, triglycerides; LBP, lipopolysaccharide binding protein. ^*^ *p* < 0.05 compared to baseline values.

### 3.2 Effect of a Fructose‐ and Glucose‐Enriched Diet, Respectively, on Metabolic Parameters

**Figure 2:**
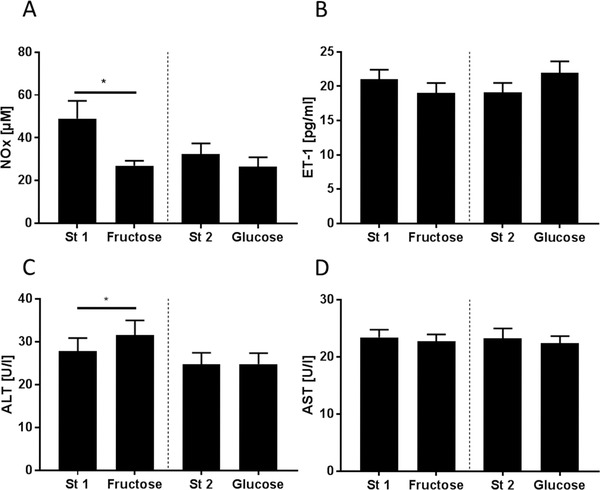
Plasma A) total nitrate/nitrite (NOx) and B) endothelin (ET)‐1 concentrations as well as serum C) alanine aminotransferase (ALT) and D) aspartate aminotransferase (AST) activity after consuming a standardized diet (St 1 and St 2) for 4 days and after a high‐fructose (Fructose) and high‐glucose (Glucose) diet for 3 days, respectively, in normal weight healthy adults. ^*^ *p* < 0.05 compared to standard diet.

Neither fasting insulin nor glucose or triglyceride serum levels were changed after either monosaccharide intervention (**Table** 4). Also, while uric acid levels in serum were not affected by the high fructose intake the high glucose diet led to a significant decrease. However, while systolic and diastolic blood pressure also remained unaffected by the different diets (Table 4), plasma NOx levels shown to be indicative of vascular endothelial dysfunction^15^ were significantly lower after subjects had consumed the fructose‐enriched diet for 3 days (≈–47%). A similar drop in NOx levels was not found after subjects had eaten the glucose‐enriched diet (see **Figure** 2). However, plasma ET‐1 levels were not affected by either diet (Figure 2).

**Table 4:** Anthropometric and metabolic parameters after consumption of standard diet and a high fructose or high glucose diet, respectively

Parameter	Standard diet 1	Fructose‐ rich diet	Standard diet 2	Glucose‐rich diet
Weight (kg)	66.5 ± 3.3	66.2 ± 3.3	66.5 ± 3.2	66.4 ± 3.4
Waist circumference (cm)	71.3 ± 1.7	71.3 ± 1.6	71.9 ± 1.7	71.2 ± 1.6
BMI (kg m^–^²)	22.0 ± 0.7	21.8 ± 0.7	22.0 ± 0.6	21.9 ± 0.7
Systolic blood pressure (mmHg)	119.9 ± 1.6	121.3 ± 2.4	122.2 ± 1.7	120.2 ± 2.0
Diastolic blood pressure (mmHg)	68.7 ± 2.9	68.7 ± 2.6	67.8 ± 2.6	68.3 3.1
TAG (mg dL^–1^)	105.8 ± 14.3	110.3 ± 10.5	106.4 ± 12.6	101.5 ± 11.7
Fasting glucose (mg dL^–1^)	90.1 ± 1.6	90.2 ± 1.8	89.7 ± 1.8	91.8 ± 1.6
Insulin (µIU mL^–1^)	12.0 ± 1.1	11.6 ± 1.1	12.2 ± 1.5	13.4 ± 1.3
Uric acid (mg dL^–1^)	5.0 ± 0.3	4.6 ± 0.2	5.0 ± 0.3	4.5 ± 0.2^*^
d‐Lactate (µm)	19.7 ± 1.0	19.7 ± 0.8	20.5 ± 0.7	20.2 ± 1.0
Citrulline (nmol mL^–1^)	3.1 ± 1.7	2.3 ± 1.1	1.1 ± 0.3	1.4 ± 0.7

Data are shown as mean ± SEM. ^*^ *p* < 0.05 compared to the respective standard diet. TAG, triglycerides.

Aminotransferase serum activities were within the normal range for women and men throughout the study. Still, after consuming the fructose‐enriched diet for 3 days, mean ALT activity in serum was increased significantly when compared to activity after standardization (≈+1.1‐fold). A similar effect on ALT activity in serum was not found after subjects had consumed the glucose‐enriched diet (Figure 2). Aspartate aminotransferase (AST) activity was not altered by any of the dietary interventions (Figure 2).

### 3.3 Effect of a Fructose‐ and Glucose‐Enriched diet, Respectively, on Adipokines and Pro‐Inflammatory markers

As fructose‐rich diets affect levels of adipokines and pro‐inflammatory cytokines in some animal models,^16^ we determined fasting plasma levels of leptin, adiponectin, and PAI‐1 after subjects had consumed the standard diet or the monosaccharide‐enriched diets. While mean levels of PAI‐1 increased in some subjects after consuming the fructose‐rich diet compared to the standard diet, differences did not reach the level of statistical significance owing to a large interindividual variability (≈+1.5‐fold, *p*  = 0.11; **Table** 5). Leptin and adiponection plasma concentrations were not affected by any of the diets.

**Table 5:** Inflammatory markers after consumption of standard diet and a high fructose or high glucose diet, respectively

Parameter	Standard diet 1	Fructose‐ rich diet	Standard diet 2	Glucose‐ rich diet
PAI‐1 (U mL^–1^)	5.0 ± 0.9	7.3 ± 1.5	6.5 ± 1.1	6.5 ± 1.4
Adiponectin (µg mL^–1^)	10.6 ± 1.0	10.3 ± 1.0	11.1 ± 1.3	11.1 ± 1.2
Leptin (ng mL^–1^)	3.4 ± 1.2	2.7 ± 1.0	3.7 ± 1.4	3.2 ± 1.3

Data are shown as mean ± SEM. PAI‐1, plasminogen activator inhibitor‐1.

### 3.4 Effect of a Fructose‐ and Glucose‐Enriched Diet, Respectively, on Markers of Intestinal Permeability

**Figure 3:**
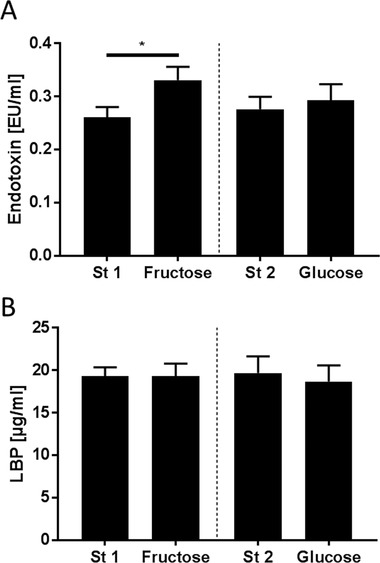
Plasma A) endotoxin and B) lipopolysaccharide binding protein (LBP) concentrations after consuming a standardized diet (St 1 and St 2) for 4 days and after a high‐fructose (Fructose) and high‐glucose (Glucose) diet for 3 days, respectively, in normal weight healthy adults. ^*^ *p* < 0.05 compared to standard diet.

Plasma endotoxin concentrations increased significantly when subjects consumed the fructose‐enriched diet for 3 days compared to the respective standard diet (≈+1.3‐fold). Similar changes were not found after the intake of the glucose‐enriched diet (**Figure** 3). Plasma concentrations of LBP were not changed after the consumption of either diet (Figure 3). Also, d‐lactate and l‐citrulline levels were not affected by the different monosaccharide‐enriched diets (Table 4).

### 3.5 Effect of the Fructose‐ and Glucose‐Enriched Diets, Respectively, on *TLR2* and *TLR4* as well as *MYD88* Expression in PBMCs

**Figure 4:**
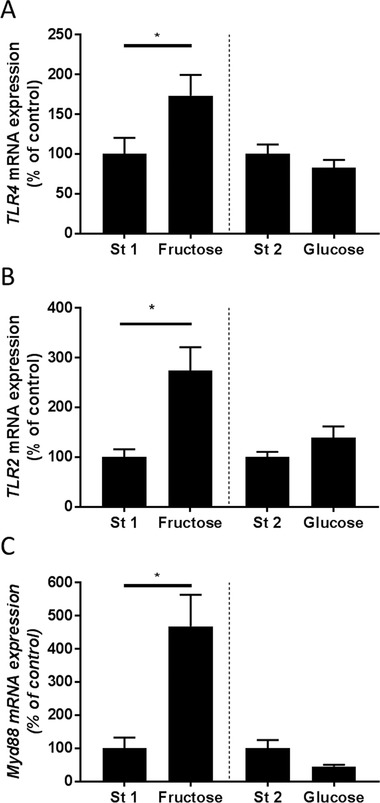
A) *TLR4*, B) *TLR2*, and C) *MYD88* mRNA expression in PBMCs in responses to a 4 day standard diet (St 1 and St 2), high fructose (Fructose), and high‐glucose diets for 3 days, respectively. MYD88, myeloid differentiation primary response gene 88; TLR, toll‐like receptor; ^*^ *p*<0.05 compared to standard diet.

Expressions of *TLR4* (≈+1.7‐fold) but also of *TLR2* (≈+2.7‐fold) and *MYD88* (≈+3.7‐fold) mRNA in PBMCs were significantly increased after subjects had consumed the fructose‐enriched diet but not after subjects received the glucose‐enriched diets as compared to standard diet (**Figure** 4).

## 4 Discussion

Along with the industrialization, life‐style including dietary pattern has markedly changed and is by now discussed to be the key risk factor for the increased prevalence of metabolic diseases (for overview see ref. [17]). However, while results of numerous studies employing rodent but also nonprimate animal models provide strong indications that sugar and herein especially fructose may be a critical factor in the development of metabolic diseases including NAFLD but also hypertension associated with vascular endothelial dysfunction (for overview see refs. [18] and [19]), studies in humans are contradictory (for overview see ref. [10]). Also, molecular mechanisms involved have not yet been fully understood. Here, after nutritionally standardizing healthy young normal weight adults for 4 days to a “healthy” diet as recommended by the German nutritional society, complex carbohydrates were isocalorically exchanged with fructose and glucose, respectively (25% of total energy intake, ≈160 g d^–1^) for 3 days to determine effects on endotoxin plasma levels as well as markers on metabolic health. While when compared to fructose intake of the general US and European population, this amount of fructose intake has to be considered as rather high,^20^ results of studies suggest that certain subpopulations in the US and Europe with high intake of fruit juices and sodas as well as sweets, might achieve comparable daily intakes of fructose.^21^ In our present study, the isocaloric exchange of complex carbohydrates with fructose (25% of total energy derived from fructose) for 3 days resulted in a significant increase of ALT activity while NOx levels in blood decreased suggesting that a short‐term intake of a fructose‐enriched diet may affect both, liver and vascular endothelial health.^6^, ^19^ Similar changes of these parameters were not found after the consumption of isocaloric amounts of glucose. Interestingly, ALT activity in blood was also significantly altered after subjects were nutritionally standardized. Indeed, while subjects were all normal weight and metabolically healthy, the “normal” diet of subjects was rather rich in saturated fat (≈40% of total energy intake derived from fat with ≈18% from saturated fatty acids), which is also suggested previously to be critical in the development of NAFLD and cardiovascular diseases.^22^ However, the nutritional standardization was also associated with a significant increase in triglyceride and fasting insulin levels. It has been shown before that altering nutritional intake towards a diet rich in carbohydrates and low in fat may lead to higher triglyceride levels^23^ and elevated postprandial insulin levels compared to diets rich in fat.^24^

The lack of changes in AST activity, endothelin‐1, and blood pressure as well as adipokines and PAI‐1 after consuming a fructose‐rich diet is in contrast to the results of some animal and some human studies^19^, ^25^ and may be attributed to the short‐term intervention in our study. Indeed, in most of these studies, the monosaccharide was consumed for several weeks.^25^, ^26^ Therefore, it cannot be ruled out that an extended consumption of fructose might also have affected these parameters in the present study. Additionally, in contrast to previous studies^25^, ^27^ showing a markedly increase in uric acid concentrations after the intake of high amounts of fructose, serum uric acid concentrations did not change after the high fructose consumption in the present study. Differences between the findings of the present study and those of others might have resulted from differences in age and gender of subjects studied. Indeed, it has been shown before that men are more often affected by gout than women and the prevalence increases with age.^28^ Additionally, the amount and duration of fructose consumption was different (200 g for 2 weeks vs mean intake of 160 g for only 3 days in the present study).

Results of several animal studies further suggest that chronic elevated fructose intake may lead to the development of NAFLD and that this at least in part results from impairments of intestinal barrier function and an increased translocation of bacterial endotoxin.^25^, ^29^ Similar relations have also been reported for fructose‐induced vascular endothelial dysfunction in rodent models.^30^ In the present study, the isocaloric exchange of complex carbohydrates of the standard diet with fructose but not glucose was associated with a significant increase of plasma endotoxin concentrations; however, LBP, l‐citrulline, and d‐lactate levels in plasma all shown before to be indicative of an impaired intestinal barrier function^31^ were not altered under either monosaccharide intervention. The latter might have resulted from the short intervention period but also from severity of the trigger, e.g., a monosaccharide being a “normal” compound of human diet. For instance, it has been shown before that even under severe disease stages like sepsis associated with significant increases in endotoxin it may take even days until LBP levels are increased.^32^ Increases of both, l‐citrulline and d‐lactate have been shown to be associated with severe impairments of intestinal barrier function like inflammatory bowel disease 33 while not being changed in early stages of NAFLD31c further suggesting that these markers might be indicative of more severe intestinal barrier dysfunction.

In support of the findings that a fructose‐enriched diet promotes the gut translocation of bacterial endotoxin, expression of *TLR4* and *MYD88* mRNA in PBMC were significantly higher after the 3 days of consuming the fructose‐enriched diet when compared to expression levels after subjects had consumed the standard diet. Furthermore, the finding that mRNA expression of *TLR2* was also significantly higher after subjects consumed the fructose‐enriched diet for 3 days suggests that not only translocation of bacterial endotoxin was increased but maybe intestinal permeability per se was altered. Indeed, expression of *TLR2* being a ligand for toxins of Gram‐positive bacteria also shown before to be induced in liver tissue in patients with NAFLD and to be associated with an increased intestinal permeability^34^ was also significantly higher in PBMCs after subjects had consumed fructose. Taken together, these results suggest that even after a short‐term ingestion of larger amounts of fructose intestinal barrier function is altered while similar changes are not found when identical amounts of glucose are consumed. Our results by no means rule out that an extended consumption of glucose may also impair intestinal barrier function or occur after some time.

### 4.1 Limitations

Our study is not without limitations. The major limitation is the rather small sample size of only 12 subjects and the limitation of the study to young healthy adults. Results might differ in a larger and older population. However, as both, the dietary standardization of participants and the 3 day consumption of the monosaccharide‐enriched diets required a high compliance and adherence to the study design, it was not possible to enroll more study participants. Still, despite the small sample size findings are in line with others showing effects of an excessive fructose intake on ALT activity.^35^ Furthermore, our study was not performed in a cross‐over design as we encountered several drop‐outs due to unknown fructose intolerance of the participants. To avoid late drop‐outs, we adapted the study design and all participants received the high‐fructose diet as first intervention. Another limitation of the study is the lack of a direct measurement of intestinal permeability, e.g., through a xylose test or lactose‐mannitol test.^36^ However, as these tests require urine collection over several hours and the dietary interventions were rather rigid, these additional tests would have resulted in a lower compliance decreasing the number of participants even more. Also, liver health was only assessed through surrogate markers, e.g., activity of transaminases in serum. However, based on previous studies of other groups^37^ reporting that the consumption of a high‐fructose‐ and high‐glucose‐diet (150 g d^–1^ and 25% of energy intake, respectively), when consumed for 2–4 weeks was not afflicted with hepatic fat content as assessed by magnetic resonance imaging, we did not assume any changes in our short‐term dietary trial. Furthermore, as our study was performed in a non‐clinical setting, we refrained from assessing liver status as this would have required additional examinations and time from the study participants. Moreover, it cannot be ruled out that starch derived intake of glucose varied between the interventions. Nutrient contents of the standard and intervention diets were estimated using the nutritional software EBISpro containing the German Nutrient Database (in German: Bundeslebensmittelschlüssel, also see: https://www.blsdb.de), which comprises about 10 000 foods and their nutritional values (137 constituent data per food item) but has no information available on resistant starch. However, as all foods and beverages were provided, mostly prepared and consumed in the study center and foods consumed during the interventions were kept rather similar between participants, it can be assumed that total starch intake should not have varied markedly throughout the study.

## 5 Conclusion

Taken together, results of the present study further bolster the hypothesis that dietary fructose may be critical in the development of NAFLD and vascular endothelial dysfunction in humans and that similar to the findings in animal studies^9^, ^38^ impairments of intestinal barrier function and an increased translocation of bacterial endotoxin maybe critical herein. Also in line with earlier findings in model organisms,25a results of the present study suggest that at least short‐term glucose may not have these effects on parameters related to liver and vascular endothelial as well as intestinal barrier function. Furthermore, results of the present study also suggest that changing dietary pattern only for a few days may markedly impact liver, vascular endothelial and intestinal barrier function. However, molecular mechanisms involved and especially those underlying the effects of fructose on intestinal barrier function need to be determined in future studies.

## Conflict of Interest

The authors declare no conflict of interest.
